# The intestinal parasite *Cryptosporidium* is controlled by an enterocyte intrinsic inflammasome that depends on NLRP6

**DOI:** 10.1073/pnas.2007807118

**Published:** 2020-12-28

**Authors:** Adam Sateriale, Jodi A. Gullicksrud, Julie B. Engiles, Briana I. McLeod, Emily M. Kugler, Jorge Henao-Mejia, Ting Zhou, Aaron M. Ring, Igor E. Brodsky, Christopher A. Hunter, Boris Striepen

**Affiliations:** ^a^Department of Pathobiology, School of Veterinary Medicine, University of Pennsylvania, Philadelphia, PA 19104;; ^b^Department of Pathology and Laboratory Medicine, Institute for Immunology, Perelman School of Medicine, University of Pennsylvania, Philadelphia, PA 19104;; ^c^Division of Protective Immunity, Department of Pathology and Laboratory Medicine, Children’s Hospital of Philadelphia, Philadelphia, PA 19104;; ^d^Department of Immunology, Yale School of Medicine, Yale University, New Haven, CT 06519

**Keywords:** *Cryptosporidium*, inflammasome, immunity, NLRP6, parasite

## Abstract

The apicomplexan parasite *Cryptosporidium* infects the intestinal epithelium. While infection is widespread around the world, children in resource-poor settings suffer a disproportionate disease burden. Cryptosporidiosis is a leading cause of diarrheal disease, responsible for mortality and stunted growth in children. CD4 T cells are required to resolve this infection, but powerful innate mechanisms control the parasite prior to the onset of adaptive immunity. Here, we use the natural mouse pathogen *Cryptosporidium tyzzeri* to demonstrate that the inflammasome plays a critical role in initiating this early response. Mice lacking core inflammasome components, including caspase-1 and apoptosis-associated speck-like protein, show increased parasite burden and caspase 1 deletion solely in enterocytes phenocopies whole-body knockout (KO). This response was fully functional in germfree mice and sufficient to control *Cryptosporidium* infection. Inflammasome activation leads to the release of IL-18, and mice that lack IL-18 are more susceptible to infection. Treatment of infected caspase 1 KO mice with recombinant IL-18 is remarkably efficient in rescuing parasite control. Notably, NOD-like receptor family pyrin domain containing 6 (NLRP6) was the only NLR required for innate parasite control. Taken together, these data support a model of innate recognition of *Cryptosporidium* infection through an NLRP6-dependent and enterocyte-intrinsic inflammasome that leads to the release of IL-18 required for parasite control.

## Significance

The intestinal immune system is able to control pathogens while tolerating and interpreting microbial cues from an abundant microbiome. The mechanisms of innate recognition are crucial to differentiating between pathogen and commensal in this tissue and to mounting an appropriate inflammatory response. Persistent inflammation can alter the cellular architecture and physiology of the gut and have lasting impact on the nutritional state of children who face frequent infection with certain enteric pathogens. We demonstrate that the widespread parasite *Cryptosporidium* acts as a potent trigger for an enterocyte-intrinsic inflammasome that depends on the NOD-like receptor pyrin domain-6 and results in the local release of the proinflammatory cytokine IL-18.

Globally, 9% of deaths in children under the age of 5 are directly attributed to diarrheal disease caused by infection (1). Diarrheal diseases are also an important cause of malnourishment in children, a condition that enhances susceptibility to many other infections including malaria, pneumonia, and measles, resulting in significantly higher mortality rates (2). Frequent episodes of enteric infection can also cause growth stunting and persistent developmental delay, a syndrome known as “environmental enteropathy” (3). Changes in the structure and physiology of the gut have been associated with environmental enteropathy, but the underlying immunological mechanisms are poorly understood. The apicomplexan parasite *Cryptosporidium* is an enteric pathogen that contributes to this syndrome and is a leading global cause of illness and death in young children (4, 5). The molecular mechanisms by which this parasite causes disease and long-term sequelae are largely unknown.

Until recently, in vivo studies of *Cryptosporidium* infection were limited to large animal models including calves and piglets or to mice with diminished immune function (through chemical suppression, genetic insufficiency, or use of neonates). To overcome some of the challenges associated with these studies, we recently developed a mouse model for cryptosporidiosis using *Cryptosporidium tyzzeri*, a natural pathogen of house mice with a genome >95% identical to that of the human-specific parasites *Cryptosporidium parvum* and *Cryptosporidium hominis* (6). Murine infection with *C. tyzzeri* resembles human cryptosporidiosis in location, pathology, and resolution and provides an important tool to define the host and parasite factors that determine the outcome of infection and to identify the immune mechanisms required for vaccine-mediated protection to prevent cryptosporidiosis.

While the resolution of *Cryptosporidium* infection in humans (7) and mice (6, 8) depends on T cell-mediated adaptive immunity, there is a potent innate mechanism of resistance that depends on the early production of IFN-γ, which restricts the initial replication of this pathogen (6, 9, 10). The cytokines IL-12 and IL-18 have been implicated in lymphocyte production of IFN-γ, but the mechanism of innate recognition of *Cryptosporidium* that leads to this early cytokine response remains poorly defined. The inflammasome is a multiprotein complex that plays a key role in innate immune responses to viral, bacterial, and protozoan pathogens (11, 12). Distinct members of the NOD-like receptor (NLR) family act as cytosolic sensors that enable innate recognition of a wide variety of intracellular molecules associated with infection. In response to specific signals, some NLRs were shown to oligomerize and recruit the adaptor protein apoptosis-associated speck-like protein (ASC), which in turn recruits and activates the proprotease caspase-1 (11). Active caspase-1 cleaves and activates its targets, which include procytokines of the IL-1 family, as well as pore-forming molecules such as gasdermin D. Together, these activities lead to the release of active IL-1β and IL-18 and an inflammatory form of cell death known as pyroptosis. For *C. parvum*, recent studies by McNair and colleagues showed that neonatal mice that lacked caspase-1 or ASC were more susceptible to infection, providing evidence for the involvement of the inflammasome in cryptosporidiosis (13).

The role of the inflammasome in innate recognition and resistance to infection has been studied primarily in innate immune cells, particularly in macrophages. However, *Cryptosporidium* invades and replicates within intestinal epithelial cells, which raises the question of which inflammasome components contribute to cell-intrinsic sensing of this infection. Using a natural host-adapted *Cryptosporidium*, we demonstrate that control of this organism relies on an epithelial cell-intrinsic caspase-1. This response was exclusively dependent on NOD-like receptor 6 (NLRP6) and required gasdermin D and IL-18. Moreover, exogenous IL-18 rescued the ability of caspase-1–deficient mice to control *Cryptosporidium*, indicating that the role of inflammasome activation is to promote the release of IL-18. Collectively, these findings implicate epithelial cell-intrinsic NLRP6/caspase-1–mediated secretion of IL-18 as a key mechanism in the control of *Cryptosporidium* and demonstrate that NLRP6 contributes to the control of an enteric parasite.

## Results and Discussion

### Mice Lacking Core Components of the Inflammasome Are Susceptible to Acute *C. tyzzeri* Infection.

**Fig. 1.**
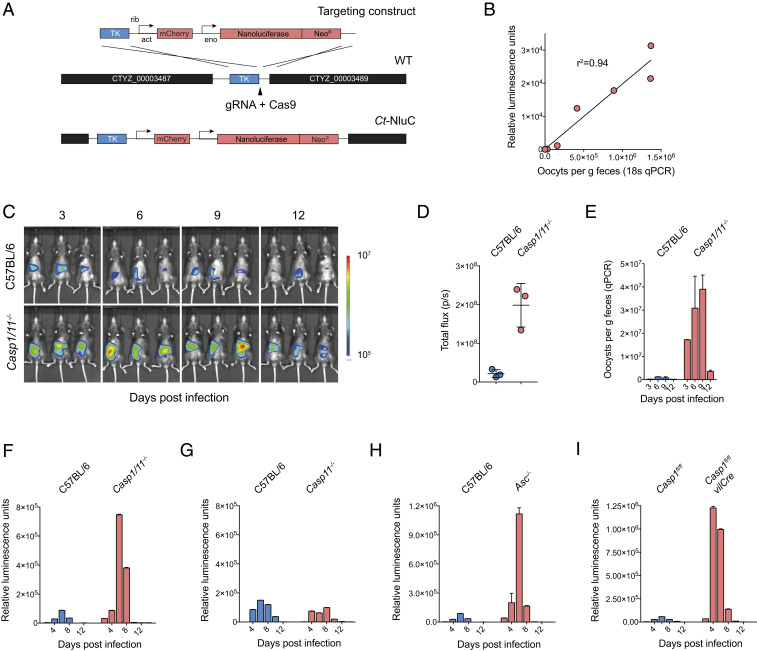
Mice deficient in core components of the inflammasome have a higher parasite burden. (*A*) Schematic map of the strategy used to derive the Ct-NluC strain. (*B*) Graph of parasite burden measured by qPCR (*x* axis) and luminescence (*y* axis). Each measurement represents the average of three technical replicates from the same mouse fecal sample, and the linear relationship has an R^2^ value of 0.94. (*C*–*E*) C57BL/6 (WT) and *Casp1/11*^*−/−*^ mice were infected with 50,000 Ct-FluC oocysts each, and parasite burden was measured by whole-animal imaging (*C* and *D*) and qPCR (*E*). Quantitative PCR was performed on pooled collected samples with two technical replicates; mean ± SD and *n* = 6 mice in total. (*F*–*H*) Parasite shedding in C57BL/6 (WT) versus *Casp1/11*^*−/−*^ (*F*), *Casp11*^*−/−*^ (*G*), and *Asc*^*−/−*^ (*H*) mice infected with 50,000 Ct-NluC oocysts per mouse. Fecal luminescence measurements are from pooled cage samples shown as mean ± SD from two technical replicates. (*I*) Comparative infection of *Casp1*^*fl/fl*^ mice and *Casp1*^*fl/fl*^ -*vilCRE* mice that are deficient in caspase 1 specifically in their intestinal epithelial cells. Mice were infected with 50,000 Ct-NluC parasites, and fecal luminescence is from pooled collections shown as mean ± SD from two technical replicates per time point. Data are from two separate experiments, each with *n* = 8 mice. For *F* and *G*, *n* = 8 mice. For *H*, *n* = 7 mice in total. Samples in *F*–*I* are reported for every 2 d, but the *x* axis is abbreviated to 4-d increments for clarity.

To determine the role of the inflammasome in innate responses to *C. tyzzeri*, we infected knockout mice lacking inflammasome activity with two transgenic *C. tyzzeri* strains. The first strain expresses a red-shifted luciferase (strain Ct-FluC) to visualize parasite burden in the tissue, which we recently reported (6). The second was a newly derived strain (CT-NluC), which allowed rapid measurement of fecal parasite burden. For this new strain, we used Cas9-directed homologous repair (14) to insert a cassette next to the thymidine kinase locus (*Methods*) to constitutively express Nanoluciferase and the fluorescent protein mCherry (Fig. 1*A*). The Nanoluciferase activity measured in the feces of mice infected with these parasites is tightly correlated in a linear fashion with levels of *Cryptosporidium*-specific DNA measured by qPCR from the same sample. Thus, Nanoluciferase activity serves as a reliable indicator of parasite shedding (Fig. 1*B*).

Both transgenic strains readily infect wild-type (WT) C57BL/6 mice with peak parasite burden occurring at day 6 of infection and resolution after 10 d. In contrast, *Casp1/11*^−/−^ mice demonstrated a much higher early burden (Fig. 1 *C*–*F*) regardless of parasite strain or measurement used. During this acute phase, whole-body luminescence measured from *Casp1/11*^*−/−*^ mice was nearly 10-fold higher than that of matched C57BL/6 controls (Fig. 1*D*). Quantitative PCR using *Cryptosporidium*-specific primers on fecal pellets collected from WT and *Casp1/11*^*−/−*^ mice revealed higher shedding (Fig. 1*E*), as did fecal luciferase measurements from *Casp1/11*^−/−^ mice infected with the Ct-NluC strain (Fig. 1*F*). These results are consistent with experiments that used *C. parvum* (13) and suggest that the inflammasome is broadly required for resistance to diverse *Cryptosporidium* species.

*Casp1/11*^*−/−*^ mice lack two caspases with distinct but overlapping functions (15). Therefore, to determine whether caspase-1 or -11 (or both) were responsible for the differences in parasite burden observed, we measured infection in mice solely lacking caspase 11 (15). In contrast to the *Casp1/11*^*−/−*^ mice, *Casp11*^*−/−*^ mice were no more susceptible to infection than control mice, indicating that caspase-1 is sufficient to provide innate resistance to cryptosporidiosis and suggesting that the canonical inflammasome is likely involved (Fig. 1*G*). ASC is a core component of canonical inflammasome complexes that contain an NLR and caspase-1. ASC contains an N-terminal pyrin domain and a C-terminal caspase activation and recruitment domain and functions as an adaptor that bridges caspases-1 and the pathogen/danger-sensing NOD-like receptors. We note that *Asc*^*−/−*^ mice had an increased parasite burden during the acute phase of infection but eventually were able to control *Cryptosporidium*, similarly to *Casp1/11*^*−/−*^ mice (Fig. 1*H*).

We next sought to further define the cell type in which inflammasome activation was required in vivo with the consideration that *Cryptosporidium* specifically infects the epithelial cell layer within the intestine. To specifically isolate these cells, we used *Casp1*^*fl/fl*^-*vilCRE* mice that lack caspase 1 only in their enterocyte populations (16). Importantly, these mice demonstrated an 18.7-fold increase in parasite burden over the unfloxed *Casp1*^*fl/fl*^ strain. This increase is indistinguishable from that observed in whole-body *Casp1/11*^*−/−*^-deficient mice, suggesting that caspase-1 activation in enterocytes is required to enable early control of *Cryptosporidium* in vivo.

**Fig. 2.**
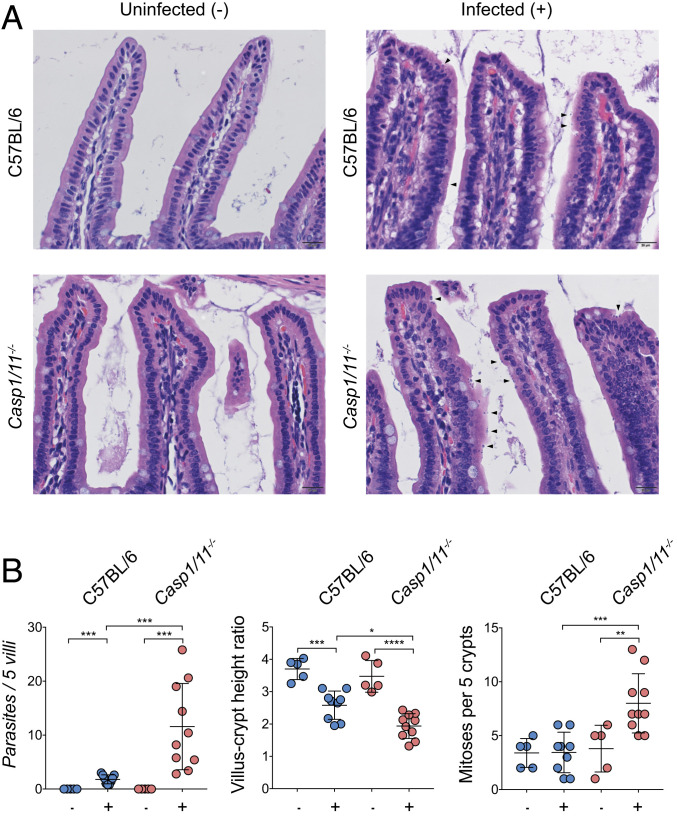
Loss of the inflammasome leads to more severe intestinal pathology. C57BL/6 (WT) mice and *Casp1/11*^*−*/-^ mice were infected with 50,000 Ct oocysts each, followed by histological examination of the small intestine on day 5 post infection. (*A*) Representative images of infected and uninfected mice with parasites highlighted by arrowheads. (*B*) Histological scoring from a veterinary pathologist; *n* = 30, with the scoring from each mouse represented as a single measurement. Mean ± SD with significance determined using one-way ANOVA and Tukey’s multiple comparisons test (*P < 0.05, **P < 0.005, ***P < 0.0005, and ****P < 0.00005).

In order to characterize the physiological impact of inflammasome deficiency on the local response to *Cryptosporidium* in the gut, we conducted histological analysis using a previously established grading rubric (6). At day 5 post infection, the small intestines were removed from uninfected and infected mice, and hematoxylin-and-eosin–stained sections were evaluated by microscopy (Fig. 2*A*). At baseline, the structure and organization of the villi of uninfected *Casp1/11*^*−/−*^ mice and WT C57BL/6 controls were not significantly different, and there were no overt signs that loss of the inflammasome resulted in changes in intestinal structure. In response to infection, WT mice showed a reduction in the villus-to-crypt ratio, a marker of intestinal inflammation, but no increase in the mitotic index in the crypts (Fig. 2 *A* and *B*). In contrast, parasites were more readily detected in *Casp1/11*^*−/−*^ mice (arrowheads), and these mice demonstrated a lower villus-to-crypt height ratio and an increase in crypt mitotic rate, indicating a greater level of tissue turnover (Fig. 2*B*).

### IL-18 Release Is Required for the Innate Control of *C. tyzzeri*.

**Fig. 3.**
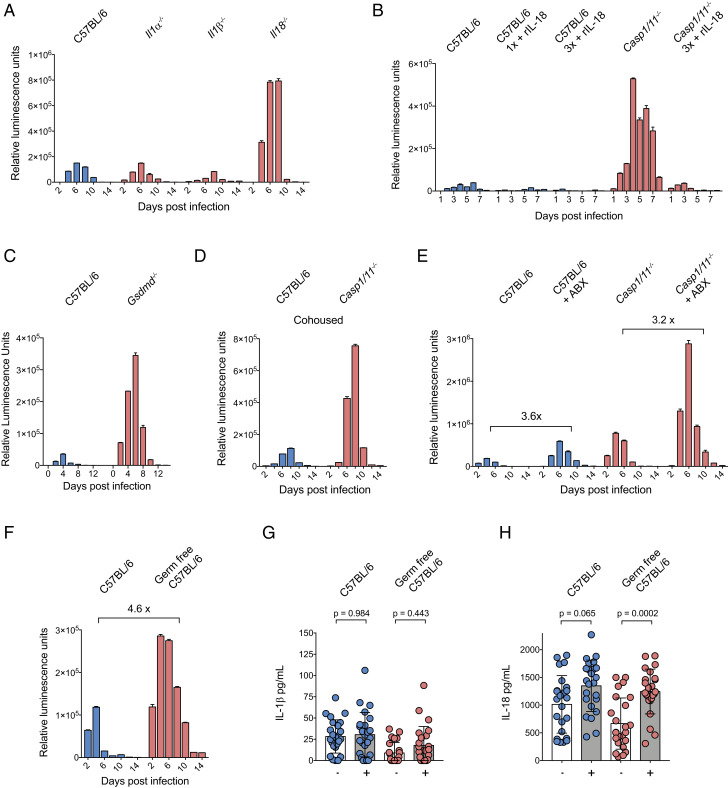
*Cryptosporidium* inflammasome activation is independent of the microbiome and drives IL-18 secretion. (*A*) Parasite burden from C57BL/6 (WT), *Il1α* ^*−/−*^, *Il1β* ^*−/−*^, and *Il18*^*−/−*^ mice and (*C*) C57BL/6 and *Gsdmd*^*−/−*^ mice infected with 50,000 Ct-NluC oocysts per mouse. Four mice per group; *n* = 16 or *n* = 8 mice total, respectively. Fecal luminescence was measured from pooled cage samples, and mean ± SD is shown from two technical measurements per time point. (*B*) Parasite burden from rIL-18–treated and untreated C57BL/6 (WT) and *Casp1/11*^*−/−*^ mice. Mice were infected with 50,000 Ct-NluC parasites each, and 2 μg of rIL-18 was administered by intraperitoneal injection every second day starting on day 1 post infection. Fecal luminescence was measured from pooled cage samples, and mean ± SD is shown from two technical measurements per time point. Four mice per group with *n* = 20 mice total. One group received a single rIL-18 injection which alone decreased parasite burden by 64.4%. (*D*) C57BL/6 and *Casp1/11*^*−/−*^ mice were cohoused for 2 wk prior to infection with 50,000 Ct-NluC oocytes per mouse. Fecal luminescence shown as mean ± SD from two technical replicates per time point; *n* = 12 from two separate experiments. (*E*) C57BL/6 and *Casp1/11*^−/−^ mice were treated for 1 wk with an antibiotic mixture (*Methods*) ad libitum in their cage water and then infected with 50,000 Ct-NluC oocysts per mouse. Fecal luminescence shown as mean ± SD from two technical replicates per time point; *n* = 16 from one experiment. (*F*) C57BL/6 germfree mice and matched controls were infected with 50,000 Ct-Nluc parasites by gavage, and fecal luminescence was measured from pooled cage fecal collections. Fecal luminescence shown as mean ± SD from two technical replicates per time point; *n* = 8 from one experiment. (*G* and *H*) ELISA measurements of IL-1β and IL-18 and from intestinal segments of infected and uninfected mice. Mice from *G* and *H* were a part of a cohort; therefore, significance was determined using one-way ANOVA with Tukey’s multiple comparisons test. Four mice per condition with *n* = 16 mice total and six intestinal segments harvested per mouse on day 5 post infection. Each mouse was infected with 50,000 Ct-NluC oocysts. Samples in *A* and *C*–*F* are reported for every 2 d, but the *x* axis is abbreviated to 4-d increments for clarity.

Inflammasome activation can lead to the processing and release of the cytokines IL-1β and IL-18. To determine the contribution of IL-1 family members in resistance to *C. tyzzeri*, mice that lack IL-1α, IL-1β, or IL-18 were infected with *C. tyzzeri*, and parasite burden was measured. WT mice and those lacking IL-1β or IL-1α showed comparable burden; however, IL-18–deficient mice showed a marked increase in acute parasite burden. Similar to *Casp1/11*^*−/−*^ mice, *Il18*^*−/−*^ mice were able to eventually control the infection (Fig. 3*A*). The kinetics and magnitude of infection in *Il18*^*−/−*^ mice closely resembled that of mice lacking core inflammasome components, suggesting that inflammasome-dependent IL-18 release is an important effector mechanism required for innate resistance to *Cryptosporidium*. To test this hypothesis, we next asked whether treatment with recombinant IL-18 (rIL-18) would rescue innate immune resistance in *Casp1/11*^*−/−*^ mice. We used a version of recombinant IL-18 that is not susceptible to inhibition by IL-18–binding protein (17). A dose of 2 μg of rIL-18 was administered intraperitoneally to WT or *Casp1/11*^*−/−*^ mice every 2 d following infection. In WT mice, this treatment resulted in enhanced protection. Notably, in *Casp1/11*^*−/−*^ mice, rIL-18 treatment was sufficient to rescue innate control of *Cryptosporidium* (Fig. 3*B*).

Like other members of the IL-1 cytokine family, IL-18 lacks an N-terminal signal peptide typically associated with export via the canonical secretory pathway. Instead, it is secreted via an unconventional pathway that requires processing by caspase-1 and the pore-forming activity of the gasdermin family of proteins (18). To test whether gasdermin D (GSDMD) might contribute to control of *Cryptosporidium*, *Gsdmd*^*−/−*^ and WT mice were infected and parasite burden was measured. In these experiments, *Gsdmd*^*−/−*^ mice had a 10-fold increase in parasite burden compared to WT controls (Fig. 3*C*), an increase that is comparable to transgenic mice deficient in inflammasome core components and those lacking IL-18. Together, these experiments support the model that inflammasome-deficient mice lack IL-18 activation and release through GSDMD, leading to increased susceptibility to *Cryptosporidium* infection.

### *Cryptosporidium* Inflammasome Activation Does Not Require the Intestinal Microbiome.

**Fig. 4.**
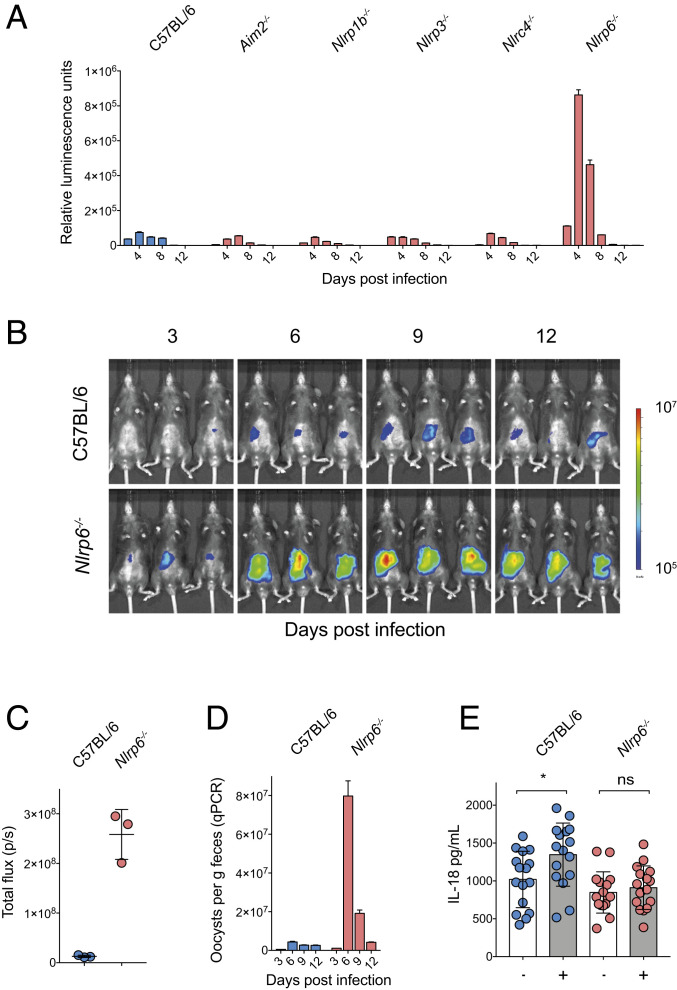
NLRP6 is responsible for resistance to *Cryptosporidium* infection. (*A*) Parasite shedding from a comparative infection of C57BL/6, *Aim2*^*−*/-^, *Nlrp3*^*−/−*^, *Nlrp1b*^*−/−*^, *Nlrc4*^*−/−*^, and *Nlrp6*^*−/−*^ mice infected with 50,000 Ct-Nluc oocysts. Four mice per group with *n* = 24 mice total. Fecal luminescence was measured from pooled cage collections with mean ± SD shown from two technical replicates. Samples in *A* are reported for every 2 d, but *x* axis is abbreviated to 4-d increments for clarity. (*B*–*D*) C57BL/6 (WT) and *Nlrp6*^*−/−*^ mice were infected with 50,000 Ct-FluC oocysts, and parasite burden was measured by whole-animal imaging (*B* and *C*) and qPCR (*D*). qPCR was performed on pooled collected samples with two technical replicates; mean ± SD and *n* = 6 mice in total. (*E*) ELISA measurements of IL-18 from intestinal segments of infected and uninfected C57BL/6 and *Nlrp6*^*−/−*^ mice. Note that lack of NLRP6 ablates the increase in IL-18 production from *Cryptosporidium* infection. Significance was determined using one-way ANOVA with Tukey’s multiple comparisons test. Four mice per condition with *n* = 16 mice total and four intestinal segments harvested per mouse on day 5 post infection (*P < 0.05).

While the inflammasome can be activated in response to pathogens, exposure to the natural flora of the gut can also engage this pathway, and the lack of inflammasome components can lead to alterations in the microbiome that subsequently affect susceptibility to intestinal inflammatory insults (19). Therefore, we conducted experiments to evaluate the impact of the microbiome on inflammasome activation in the context of cryptosporidiosis. To minimize microbiome differences, *Casp1/11*^*−/−*^ mice and C57BL/6 controls were cohoused for 2 wk prior to infection. This did not affect parasite burden measurements in either strain (Fig. 3*D*), suggesting that the observed susceptibility phenotype is not driven by transferrable differences in the intestinal microbiome of these mice. Next, we treated *Casp1/11*^*−/−*^ and WT C57BL/6 mice with a mixture of broad-spectrum antibiotics prior to infection to reduce the intestinal microbiome. As previously shown, mice treated with antibiotics show an increase in the total parasite burden (6, 14) (Fig. 3*E*). However, this increase in parasite burden was inflammasome independent, as *Casp1/11*^*−/−*^ mice displayed the same increase in parasite burden from antibiotic pretreatment as C57BL/6 controls (Fig. 3*E*). Finally, we infected normal and germfree C57BL/6 mice, thereby ensuring that the only microbe present in the latter was *Cryptosporidium*. Germfree mice show higher parasite shedding than normal mice, and the increase resembles the increase associated with antibiotic treatment (4.6- and 3.6-fold, respectively) (Fig. 3 *F* and *E*). Sections of the small intestine were removed from uninfected and infected mice, and these intestinal explants were assayed for secretion of cytokines. In samples from uninfected mice, there were basal levels of IL-1β and IL-18, and, in response to infection, IL-1β levels were not altered (Fig. 3*G*) whereas levels of IL-18 increased (Figs. 3*H* and 4*E*). Infected germfree mice also demonstrated increased IL-18 secretion when compared to uninfected germfree controls (Fig. 3*H*). Taken together, these data show that, in the absence of the microbiome, *Cryptosporidium* infection alone is capable of driving processing and secretion of IL-18 in the small intestine. Furthermore, while the presence of the intestinal microbiota antagonizes *Cryptosporidium* growth, this activity appears to be inflammasome independent.

### The Enterocyte NOD-like Receptor NLRP6 Is Required to Control *Cryptosporidium*.

We next sought to discover the specific pathogen receptor(s) that detect *Cryptosporidium* infection. In the related apicomplexans *Toxoplasma* and *Plasmodium*, the NOD-like receptors NLRP1b and NLRP3, as well as the pyrin domain containing absent in melanoma protein 2 (AIM2) have been associated with parasite control (20–23). We tested a comprehensive set of mouse strains deficient in the expression of different NOD-like receptors and AIM2 for susceptibility to *C. tyzzeri*. Mice that lack AIM2, NLRP1b, NLRP3, and NLRC4 showed parasite burdens that were indistinguishable from WT mice (Fig. 4*A*). In contrast, NLRP6-deficient mice demonstrated increased parasite shedding and marked susceptibility to *Cryptosporidium* infection when compared to controls (Fig. 4*A*). To confirm that higher levels of parasite shedding in the feces coincided with an increase of parasite infection in the tissue, we next infected *Nlrp6*^*−/−*^ mice with the Ct-FLuC strain to allow for quantification of parasite burden in the gut (Fig. 4*B*). This revealed that *Nlrp6*^*−/−*^ mice had a nearly 20-fold higher tissue burden compared to WT C57BL/6 controls, yet were eventually able to control infection (Fig. 4 *C* and *D*). Finally, we tested the impact of lack of NLRP6 on intestinal IL-18 production. Comparison of uninfected and infected intestinal segments from WT or *Nlrp6*^*−/−*^ mice showed that, while infection in WT mice resulted in increased processing and secretion of IL-18, *Nlrp6*^*−/−*^ mice failed to release IL-18 at a higher level than uninfected controls (Fig. 4*E*). In conclusion, these data show that NLRP6 is the NOD-like receptor required to trigger an inflammasome response that enables early control of *Cryptosporidium* replication.

## Conclusion

The inflammasome plays an important role in the ability of host cells to perceive and decode a complex variety of molecular patterns associated with infection, microbial colonization, and tissue damage. Numerous NOD-like receptors exist to detect and interpret molecules that serve as sentinels of infection and danger (11, 19). In this study, we found that many of those receptors previously implicated in the control of parasites or intestinal bacteria (20–25) are not required for resistance to *Cryptosporidium* but identify a role for NLPR6-dependent restriction of the natural mouse pathogen *C. tyzzeri*. Restriction of parasite burden is also dependent on ASC and caspase-1, and importantly, is intrinsic to enterocytes. The NOD-like receptors NLRP1b and NLRP3, as well as AIM2, have been implicated in control of the related apicomplexans *Toxoplasma* and *Plasmodium* (20–23), but were not required for control of *Cryptosporidium*. This difference likely reflects the pronounced differences in tissue and host-cell tropism among these parasites with *Cryptosporidium* being restricted to enterocytes while *Toxoplasma* and *Plasmodium* cause systemic infection of multiple cell types.

NLRP6 is a NOD-like receptor that has attracted particular interest due to its preferential expression in epithelia. Its molecular activity as a component of the inflammasome is well supported and was recently resolved in structural detail by cryo-electron microscopy (26). However, the physiological role of NLRP6 in regulating responses to microbes has been the subject of significant debate. Early studies concluded that NLRP6-deficient mice were more resistant to infection with *Listeria monocytogenes*, *Salmonella typhimurium*, and *Escherichia coli* given by intravenous or intraperitoneal routes of infection (27). Anand et al. (27) concluded that NLRP6 was a negative regulator of the inflammatory signaling processes required for clearance of these organisms. Similar observations were made with *Streptococcus aureus* in lung infection (28). This contrasts with the requirement for NLRP6 in the control of *Cryptosporidium* documented in our study. In the intestine, NLRP6 has been proposed to be a master regulator of the intestinal microbiome composition at homeostasis (29, 30). Further studies have shown that NLRP6 may play a more nuanced role in the maintenance of the normal intestinal microbiota (31–34). NLRP6-deficient mice are also more susceptible to oral (but not intraperitoneal) infection with mouse norovirus (35).

**Fig. 5.**
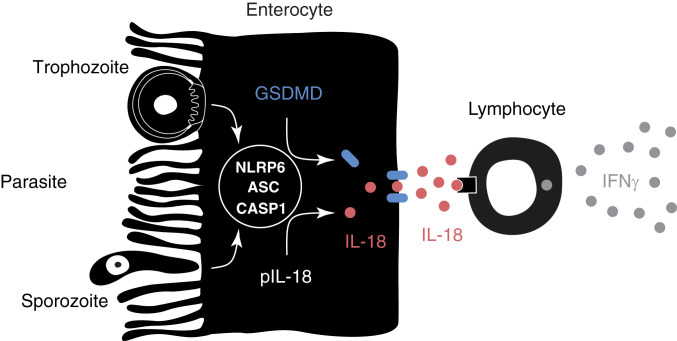
Model of inflammasome activation by *Cryptosporidium*. *Cryptosporidium* actively invades enterocytes and transforms into an intracellular replicative stage right at the brush border. In this study we demonstrate that parasite infection leads to the activation of an enterocyte intrinsic inflammasome that depends on caspase-1, ASC, and NLRP6. This activation leads to the processing and release of IL-18 (red) in a GSDMD (blue)-dependent fashion. We hypothesize that, once released, IL-18 acts on tissue resident lymphocytes to induce the production of IFNγ (gray), which restricts parasite growth by a yet-to-be-defined mechanism.

At this point, the pattern by which NLRP6 detects *Cryptosporidium* infection is unknown, but a variety of molecular patterns has been suggested to be recognized by NLRP6 in other experimental settings. NLRP6 could detect *Cryptosporidium* directly within infected cells (Fig. 5) by binding to a parasite-specific pathogen-associated molecular pattern and initiate the downstream cascade that culminates in IL-18 release. Viral RNA has been suggested as a NLRP6 trigger (35), and it is thus important to note that *Cryptosporidium* carries its own RNA virus in significant quantity (36). Specialized polyglycans, lipids, and polyketones produced by specific lifecycle stages for the complex wall of the parasite oocyst could equally serve as prominent microbial patterns (37). Lipoteichoic acid, a component of the Gram-positive bacterial cell wall, can bind NLRP6 and lead to caspase-11–mediated inflammasome activation in macrophages (38). Similarly, damage associated with parasite replication might lead to indirect NLRP6 activity within neighboring epithelial cells. The ability of microbiome-derived metabolites to activate NLRP6 may also be relevant (39, 40); however, we observed inflammasome activation in the absence of the microbiome. Establishing whether NLRP6 recognizes a *Cryptosporidium* pattern and defines its molecular identity will be an important step toward understanding how enterocytes initiate the development of innate immunity to this parasite.

One consequence of inflammasome activation by *Cryptosporidium* is the release of IL-18 and we (Fig. 3) and others (41, 42) have demonstrated that IL-18 is an important mediator of innate control for *Cryptosporidium*. While IL-18 is also known to induce IFNγ production which has a pivotal role in the innate and adaptive control of *Cryptosporidium* in mice and humans (6, 7, 10, 43, 44), the cells that recognize IL-18 and produce IFNγ remain to be fully defined. The receptors for IL-18 are expressed widely throughout the gut, and innate lymphocytes, T cells, and dendritic cells are reported to express the proinflammatory coreceptor IL-18Rβ that may responsible for driving IFNγ expression in response to *Cryptosporidium* (45). Recent studies with enteric *Salmonella* infections concluded that enterocyte IL-18 was not required for bacterial control, but rather that neuronal IL-18 was important and that expression of NLRP6 and ASC in neurons was likely involved in this response (46, 47). In contrast, based on in vitro studies, McNair and colleagues (13) suggested that dendritic cells may be a relevant source of IL-18 for *C. parvum*. Significant gaps remain in our understanding of the cellular source of IL-18 during *Cryptosporidium* infection and its ability to promote innate production of IFNγ and whether it is required for tissue repair independent of IFNγ (48, 49). Regardless, the finding that enterocyte expression of caspase 1 is critical for resistance highlights that enterocytes have an important role in recognition of this enteric pathogen, yet this finding does not exclude potential additional contributions by other cell types. Recent advances in the development of stem-cell–derived experimental models for *Cryptosporidium* (6, 50, 51) now make it possible to address questions about how enterocytes recognize and perhaps even limit parasite replication.

## Methods

### Mouse Models of Infection.

All protocols for animal care were approved by the Institutional Animal Care and Use Committee of the University of Pennsylvania. Unless otherwise specified, mice were 4 to 12 wk of age at the time of experimentation. While age and sex were controlled for each experiment, male and female mice were used indiscriminately for experiments as we have not seen a difference in infection. C57BL/6, *Casp1/11*^*−/−*^, *Asc*^*−/−*^, *Casp11*^*−/−*^, *Aim2*^*−/−*^, *Nlrp1b*^*−/−*^, *Nlrp3*^*−/−*^, and *Il18*^*−/−*^ mice were purchased from Jackson Laboratories. *Nlrc4*^*−/−*^, *Il1α*^*−/−*^, and *Il1β* ^*−/−*^ mice were provided by Sunny Shin, University of Pennsylvania, Philadelphia, PA. *Nlrp6*^*−/−*^ mice were obtained from Maayan Levy, University of Pennsylvania, Philadelphia, PA, but originated from the laboratory of Richard Flavell, Yale University, New Haven, CT. *Casp1*^*fl/fl*^ and *Casp1*^*fl/fl*^*-vilCRE* mice were bred in-house.

### Parasite Strains.

*C. tyzzeri* used in this experiment were originally isolated from wild house mice in Athens, GA (6), and propagated in *Ifng*^*−/−*^ mice initially obtained from Jackson Laboratories and bred in-house. Parasites were purified using sucrose flotation and cesium chloride gradient centrifugation.

### Generation of Transgenic Parasites.

Transgenic parasites were generated using CRISPR-directed homologous recombination and described in detail (14, 52). Briefly, oocysts were induced to excyst through treatment with sodium taurodeoxycholate and incubation at 37 °C. Excysted sporozoites were transfected with vectors to drive production of Cas9 and a parasite-specific guide RNA (gRNA) and another containing the desired genetic manipulation flanked by DNA homologous to the regions surrounding the gRNA cut site. Parasites were then used to infect *Ifng*^*−/−*^ mice by oral gavage, which was preceded by oral gavage with sodium bicarbonate to neutralize stomach acid and maintain viability of transfected sporozoites. After 24 h, parasites were selected for by treating infected mice with paromomycin (16 mg/mL ad libitum in cage water).

### Measuring Parasite Burden.

For parasites expressing Nanoluciferase, 20 mg of collected fecal sample was disrupted in 1 mL of Nanoluciferase lysis buffer by 1 min of vortexing with glass beads. Samples were centrifuged briefly at 10,000 x *g* to pellet debris, and 100 μL of supernatant was moved to a 96-well round bottom plate (Corning). A total of 100 μL from a 1:50 mixture of Nanoluciferase substrate:buffer (Promega) was then added to each sample. Luminescence was recorded using a ProMax plate reader with standard settings (Promega).

To measure parasite DNA in feces, DNA was purified from 100 mg of collected fecal samples using a Quick-DNA Fecal/Soil Microbe Kit (Zymo Research). We then used a *Cryptosporidium*-specific probe and primer set (*SI Appendix*, Table S1) and SsoAdvanced Universal Probes Supermix (BioRad) was used with a ViiA 7 Real-Time System (Thermo Fisher Scientific) to perform qPCR.

To quantify infection with *Ct*FLuc parasites, mice were injected with D-luciferin subcutaneously (Gold Biotechnology) at 125 mg/kg and then anesthetized in an induction chamber using 2.5% isoflurane. After 5 min, mice were moved into an IVIS Lumina II instrument (Caliper Life Sciences), and luminescence was measured using 5 min exposure, medium binning, and 1/16 F-stop.

### Histology of Infected Tissue.

Mice were killed at day 5 post infection, and the small intestine was dissected and rinsed in cold phosphate-buffered saline (PBS). This intestine was flushed with 10% neutral buffered formalin (Sigma) and then “Swiss-rolled” and fixed overnight. Fixed samples were paraffin-embedded, sectioned, and stained with hematoxylin and eosin. Slides were evaluated by a board-certified veterinary pathologist in a blinded fashion and scored using a previously establish matrix (6). Five consecutive and complete villus-crypt structures, meaning villus and crypt lumens within the same plane of section, were selected for scoring.

### Measurement of IL-18 and IL-1b from Mouse Intestinal Segments.

On day 5 post infection, mice were killed, and their small intestine was dissected and opened length-wise. Intestinal segments 1 cm in diameter were then harvested, rinsed in PBS, and moved to a 24-well plate (Corning) filled with 200 μL RPMI with antibiotics and 10% fetal bovine serum. Segments were incubated at 37 °C for 12 h, and then 50 μL of the supernatant was used for IL-18 and IL-1β detection by enzyme-linked immunosorbent assay (ELISA) according to the manufacturer’s protocols (Thermo Fisher Scientific).

## Supplementary Material

Supplementary File

## Data Availability

All study data are included in the article and *SI Appendix*.
