# Supplementary material for: The intestinal parasite Cryptosporidium is controlled by an enterocyte intrinsic inflammasome that depends on NLRP6
Source: Proc Natl Acad Sci U S A. 2020 Dec 28;118(2):e2007807118. doi: 10.1073/pnas.2007807118 (PMC7812745; doi:10.1073/pnas.2007807118)
Supplement: Supplementary File [file pnas.2007807118.sapp.pdf]

Supplementary Information

Table S1. Primers used in this study

Purpose	Primer/Probe Name	Sequence (5' to 3')
qPCR detection of Cryptosporidium	18S-JVA-F1	ATGACGGGTAACGGGGAAT
	18S-JVA-R1	CCAATTACAAAACCAAAAAGTCC
	18S-JVA-probe	[FAM]CGCGCCTGCTGCCTTCCTTAGATG[BHQ1]
Cas9 gRNA	TKendF	GTTGGAAGAATATAATCTCTGAGG
	TKendR	AAACCCTCAGAGATTATATTCTTC
PCR diagnostics of genetically manipulated locus	TK5F1	TTCCTCTTCCTTATTATACCCACCTAA
	TK3R1	CAGCTTCTTCTCCACCTGATAATATAGTATCTGTACC
	NeoF1	CGTATGCCCGACGGTGAAGATCTTG
	ActinR1	CTATTATTACTGTTTATACCAACTCATTCTGAAG
